# Efficient reconstruction of full-length RNA isoforms using ISAtools and large-scale PacBio circular consensus sequencing data

**DOI:** 10.1093/bib/bbag403

**Published:** 2026-07-30

**Authors:** Hu Chen, Yu-Chen Zhang, Qi Dai, Zhuo-Xing Shi

**Affiliations:** College of Life Science and Medicine, Zhejiang Sci-Tech University, Second Street 928, Qiantang District, Hangzhou 310018, China; State Key Laboratory of Ophthalmology, Zhongshan Ophthalmic Center, Sun Yat-sen University, Guangdong Provincial Key Laboratory of Ophthalmology and Visual Science, Guangdong Provincial Clinical Research Center for Ocular Diseases, No. 7 Jinsui Road, Tianhe District, Guangzhou 510060, China; College of Life Science and Medicine, Zhejiang Sci-Tech University, Second Street 928, Qiantang District, Hangzhou 310018, China; College of Life Science and Medicine, Zhejiang Sci-Tech University, Second Street 928, Qiantang District, Hangzhou 310018, China; State Key Laboratory of Ophthalmology, Zhongshan Ophthalmic Center, Sun Yat-sen University, Guangdong Provincial Key Laboratory of Ophthalmology and Visual Science, Guangdong Provincial Clinical Research Center for Ocular Diseases, No. 7 Jinsui Road, Tianhe District, Guangzhou 510060, China

**Keywords:** PacBio CCS, long-read RNA sequencing, isoform reconstruction, splice site chain, transcript boundary detection

## Abstract

Accurate reconstruction and quantification of full-length RNA isoforms remain challenging in long-read RNA sequencing due to sequencing artifacts, complex splicing, and incomplete annotations. Although Pacific Biosciences circular consensus sequencing (PacBio CCS) provides high-fidelity long reads, scalable and annotation-flexible analysis frameworks remain limited. Here, we present ISAtools, an efficient framework specifically designed for PacBio CCS data. ISAtools introduces a splice site chain representation that unifies read alignments and transcript annotations into a compact format for scalable isoform reconstruction. It further integrates unsupervised density-based clustering for transcription start and end site detection and a truncation-aware quantification strategy. Benchmarking on simulated, SIRV spike-in controls, and biological datasets shows that ISAtools accurately reconstructs both annotated and novel isoforms with reliable splice structures and transcript boundaries while maintaining high computational efficiency. ISAtools processes up to 80 million reads in ~21 min using ~6 GB memory and scales to nearly 1 billion reads in under 4 h with ~8 GB memory, supporting large-scale PacBio Iso-Seq studies.

## Introduction

Long-read RNA sequencing has transformed transcriptome analysis by enabling direct observation of full-length RNA isoforms, overcoming the inherent limitations of short-read assembly for isoform reconstruction [1–5]. Among available platforms, Pacific Biosciences (PacBio) and Oxford Nanopore Technologies (ONT) offer complementary advantages: PacBio provides higher single-read accuracy (>99%), whereas ONT offers greater sequencing throughput [6–8]. Despite these advantages, accurate isoform identification remains challenging due to cDNA fragmentation, residual sequencing errors, and artifacts introduced during library construction [6, 9]. Benchmarking efforts from the Long-read RNA-seq Genome Annotation Assessment Project (LRGASP) have consistently shown that PacBio circular consensus sequencing (CCS) data achieve superior isoform reconstruction accuracy compared with ONT data, largely owing to their lower error rates and more reliable splice junction detection [1, 10–15].

Recent technological developments have further accelerated the adoption of PacBio long-read RNA sequencing. The introduction of the Revio system with Super Accuracy (SPRQ) chemistry has enabled the generation of hundreds of millions of high-fidelity long reads per run at substantially reduced cost [16], with continued improvements in sequencing chemistry (e.g. SPRQ-Nx) and SMRT Cell utilization expected to further increase throughput [17]. As a result, large-scale and high-depth PacBio Iso-Seq datasets are becoming increasingly accessible. However, this rapid expansion exposes critical computational challenges. Existing isoform reconstruction tools are often not optimized for the combination of high sequence accuracy, read truncation artifacts, and extreme sequencing depth characteristic of PacBio CCS data, leading to limitations in scalability, memory consumption, and runtime when applied to large datasets.

Several computational frameworks have been developed for long-read isoform reconstruction using distinct algorithmic strategies. IsoQuant constructs intron graphs and performs alignment-based error correction, demonstrating strong performance on error-prone long-read data [18]. Bambu infers junction chains, models novel isoform discovery rates to build context-specific references, and applies an expectation–maximization framework for isoform quantification [19]. Mandalorion reconstructs high-confidence isoforms by clustering reads according to junction chains, followed by consensus sequence generation and stringent filtering [20]. While effective in many settings, these approaches typically rely on static reference annotations or implicitly assume near-complete transcript catalogs, limiting robustness in poorly annotated transcriptomes.

An additional and less thoroughly addressed challenge lies in the accurate identification of transcription start sites and transcription end sites (TSSs/TESs), which is essential for defining truly full-length isoforms [1]. Many existing methods prioritize splice junction reconstruction while treating transcript boundaries as secondary features or relying heavily on reference annotations. This limitation becomes particularly pronounced in high-depth long-read datasets, where both boundary precision and computational efficiency are critical for scalable analysis.

To address these specific challenges for PacBio Iso-Seq data, we developed ISAtools (**I**soform **S**equencing **A**nalysis tools), a computationally efficient framework designed for accurate isoform reconstruction under varying sequencing depths and annotation completeness. ISAtools employs a splice site chain (SSC) representation that unifies read alignments and reference annotations into a standardized text format, enabling efficient large-scale processing. Building on this representation, ISAtools applies data-driven correction strategies to mitigate systematic splicing artifacts and employs an unsupervised, density-based approach to infer transcript start and end sites directly from read termini distributions [21] without reliance on reference annotations. The framework is specifically optimized for high-fidelity yet potentially truncated PacBio CCS reads, enabling robust reconstruction of full-length isoforms across diverse experimental conditions.

Through comprehensive benchmarking on simulated datasets, SIRV spike-ins, and biological samples, we demonstrate that ISAtools achieves high accuracy in full-length isoform reconstruction, including splice structure detection and transcript boundary identification, while maintaining strong computational efficiency. Its robust performance under full, reduced, and no-annotation conditions highlights its suitability for large-scale transcriptomic studies, including those involving non-model organisms and emerging high-throughput PacBio sequencing platforms.

## Materials and methods

### Data simulation

Simulated PacBio long-read RNA sequencing data were generated using IsoSeqSim to evaluate isoform reconstruction and quantification performance under controlled conditions. Simulations were conducted across multiple sequencing depths to assess robustness to data scale.

To generate biologically realistic expression profiles, transcript-level abundances were first estimated from real human and mouse RNA-seq datasets using the LRGASP simulation pipeline. These abundance profiles were then used to sample transcripts for simulation. Subsets of transcripts from GENCODE v38 (human) and GENCODE vM27 (mouse) were selected to construct simulation-specific reference annotations, which served both as input for read generation and as ground truth for downstream benchmarking.

### Isoforms identification evaluation

Isoform identification accuracy was evaluated at both the splice-structure and transcript-boundary levels. All predicted isoforms with non-zero expression were included. To ensure consistent comparisons, predicted isoforms and reference annotations were converted into an SSC representation.

For simulated datasets, SSC-level accuracy was quantified using precision, recall, and F1-score based on exact matches to ground truth:


$$\mathrm{Precision}=\frac{TP}{TP+ FP},\mathrm{Recall}=\frac{TP}{TP+ FN},F1=2\cdotp \frac{\mathrm{Precision}\cdotp \mathrm{Recall}}{\mathrm{Precision}+\mathrm{Recall}}$$

where $TP$ is the number of predicted SSCs exactly matching the ground truth, $FP$ is the number of false positive SSCs, and $FN$ is the number of ground-truth SSCs missed by predictions. TSS/TES prediction accuracy was quantified using precision, recall, and F1-score, considering a predicted site correct if it lies within ±50 bp of the corresponding reference boundary.

To validate the robustness of our benchmarking framework, we compared our metrics with gffcompare [22]. SSC-level results are numerically equivalent to gffcompare’s intron-chain matches (Supplementary Fig. S11a). For TSS/TES evaluation, our method applies a stringent isoform-level linkage constraint, requiring TSS and TES to align with the same reference transcript entity. In contrast, gffcompare employs a locus-based set-matching strategy that identifies boundary matches within the collective range of a gene locus, which may allow hybrid boundary matches across different isoforms. This methodological distinction accounts for the slightly more conservative scores observed in our analysis (Supplementary Fig. S11b–d).

To assess biological support, we further examined whether predicted splice structures and transcript boundaries were directly supported by aligned full-length reads.

The ability to detect novel isoforms was evaluated using reduced-annotation (20% and 40%) simulations, in which a subset of annotated transcripts was removed and treated as novel ground truth. Predicted novel isoforms were assessed using the same SSC-based criteria.

For real biological datasets, where global ground truth is unavailable, predicted isoforms were classified using SQANTI3 with respect to GENCODE annotations. TSS and TES predictions were independently validated using experimentally derived transcription start and polyadenylation site databases (refTSS v3.1 and polyASite v2.0 databases).

### Isoforms quantification evaluation

Isoform quantification accuracy was evaluated using simulated datasets with known transcript-level expression. Only isoforms with non-zero predicted expression and splice structures present in the ground truth were included.

Expression concordance between predicted and true values was assessed using standard correlation- and error-based metrics, including Pearson’s correlation coefficient (r), Spearman’s rank correlation coefficient (ρ), concordance correlation coefficient (CCC), and normalized root mean square error (NRMSE).

### Overview of the ISAtools framework

ISAtools is a scalable, reference-free framework for full-length RNA isoform reconstruction and quantification from PacBio CCS data. Given aligned long reads as input, ISAtools represents each read using its SSC, defined as the ordered sequence of splice junction coordinates, where each junction is represented as a donor-acceptor pair (e.g. donor_1_-acceptor_1_-donor_2_-acceptor_2_). This representation captures transcript structure while abstracting away variability in read boundaries.

The workflow comprises three main steps: (i) SSC extraction and isoform-level grouping, (ii) SSC identification and TSS/TES detection, and (iii) isoform quantification. This framework enables efficient and accurate reconstruction and expression estimation of full-length isoforms without relying on reference annotations.

### S‌SC extraction and isoform-level grouping

For each read, ISAtools extracts its chromosome, strand, SSC, and alignment start and end positions. Reads sharing identical chromosome, strand, and SSC are collapsed into isoform-level clusters, while all start and end positions within each cluster are retained for downstream TSS/TES detection but do not participate in SSC identification.

Following the collapse of identical SSCs, ISAtools organizes them into discrete SSC groups based on genomic coordinates. SSCs are first indexed by the position of their proximal donor site. The algorithm then applies a 1D clustering strategy, assigning SSCs to the same group if their intronic spans—from the first donor to the terminal acceptor—overlap. This approach efficiently partitions isoforms into gene-specific clusters while preserving computational scalability for large datasets.

Within each SSC group, consensus-based refinement removes spurious junctions and minor alignment artifacts while retaining consistently supported splice structures. Splice sites are clustered within a local window (default ±10 bp), and only sites supported by a sufficient fraction of reads (default 10%) are retained. Importantly, these low-confidence structures generally result from sequencing or alignment errors rather than low expression, ensuring that true low-abundance isoforms with consistent splice patterns are preserved.

### S‌SC identification and TSS/TES detection

Within each isoform-level SSC group, a local splice graph corrects minor junction shifts, skipped microexons, and small exon length variations. SSCs that conform to the dominant splice pattern are retained as candidate isoforms, while junctions with weak relative support are filtered to enhance precision and reduce spurious predictions.

TSS and TES positions were inferred through density-based clustering of read termini to accommodate the inherent positional variability of long-read sequencing. Specifically, genomic start and end positions of aligned reads, denoted as ${x}_k^{start}$ and ${x}_k^{end}$, are independently clustered using DBSCAN:


$${C}_i^{\mathrm{TSS}}= DBSCAN\left(\left\{{x}_k^{start}\right\},\epsilon, MinPts\right)$$

$${C}_j^{\mathrm{TES}}= DBSCAN\left(\left\{{x}_k^{end}\right\},\epsilon, MinPts\right)$$

where $\epsilon$ defines the neighborhood radius and $MinPts$ specifies the minimum number of points required to form a cluster. Each cluster represents a candidate TSS or TES event, and its representative coordinate is defined as the mode of the clustered positions.

The representative coordinate of each cluster is defined by its mode, yielding candidate TSS or TES positions. To mitigate noise from truncated or degraded molecules, only clusters supported by at least one high-confidence, full-length read are retained. Candidate full-length isoforms are then reconstructed by pairing TSS and TES clusters, with a stringent “coupling constraint” that requires at least one read to span both predicted boundaries. This dual-end validation ensures high-fidelity isoform reconstruction and prevents over-inference of chimeric or incomplete transcripts.

### Isoform quantification

Candidate isoforms are quantified by enumerating all full-length reads that exactly match both the SSC and the predicted transcript boundaries. Reads corresponding to truncated or partial isoforms are proportionally redistributed to compatible full-length isoforms, ensuring that expression estimates account for incomplete coverage without inflating false positives. Optionally, reference-guided rescue can be applied to recover low-abundance isoforms that lack sufficient read support in isolation, while stringent filtering removes spurious or poorly supported predictions. Together, these procedures provide robust and accurate quantification of isoform-level expression, preserving both high- and low-abundance transcripts while minimizing artifacts arising from sequencing errors or read truncation.

### Read preprocessing

Reads were aligned using Minimap2 [23] (v2.28), and downstream isoform reconstruction was performed using ISAtools, IsoQuant (v3.6.3), Bambu (v3.8.0), and Mandalorion (v5.2.2). Transcriptome assembly was conducted using StringTie (v2.2.3), and isoform quantification was performed using Oarfish (v0.9.4). SQANTI3 (v4.6.0) was used for isoform classification and validation.

### Computational resources

All experiments were conducted under a unified computational environment with a fixed number of threads. Runtime and peak memory usage were measured using the time command. The system ran CentOS Linux 7.9.2009 on an AMD EPYC 9354 32-Core Processor with 512 GB RAM. All tools were executed using 20 threads.

## Result

### Study design and ISAtools framework

To benchmark ISAtools against leading long-read RNA-seq analysis tools with PacBio platform, we established a comprehensive evaluation framework encompassing dataset construction, metric definition, and performance profiling across multiple analytical dimensions.

**Figure 1:**
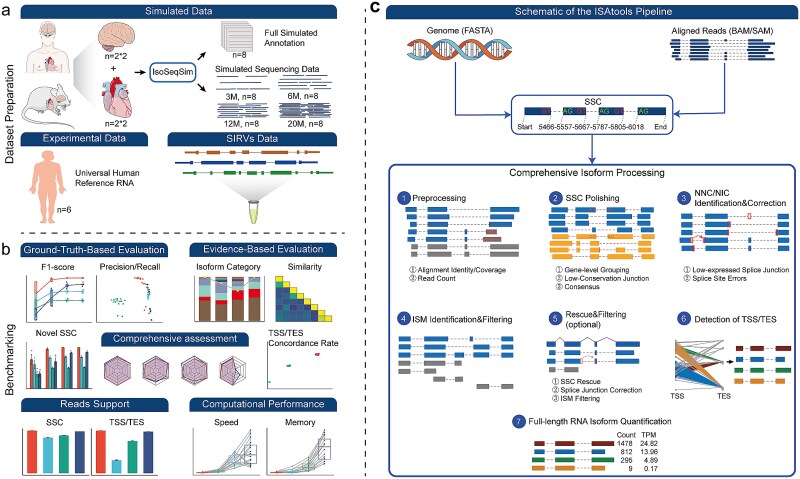
Overview of the ISAtools framework and benchmarking design; (a) dataset overview; a total of 32 simulated datasets were generated using IsoSeqSim based on PacBio error profiles, spanning multiple tissues, species, and sequencing depths, each with corresponding ground-truth annotations; experimental data included publicly available UHRR long-read RNA-seq samples (Revio platform) and SIRV spike-in controls; (b) multi-scale evaluation strategy; benchmarking covered isoform-level reconstruction accuracy on simulated and SIRV datasets, annotation concordance in real data, read-level support, computational efficiency (runtime and memory usage), and overall robustness summarized using radar plots; (c) schematic of the ISAtools pipeline; the workflow includes SSC extraction, read preprocessing, grouping and polishing of SSCs, identification and correction of artifacts, TSS/TES detection via read-end clustering, and isoform quantification with truncation-aware adjustment; see Materials and methods for details.

We assembled datasets from both simulated and real samples. The simulated dataset included human and mouse transcriptomes across two representative tissues (brain and heart), each with two biological replicates. Subsampling at four sequencing depths (3 M, 6 M, 12 M, and 20 M reads) yielded 32 distinct datasets. Real-world evaluation used the publicly available PacBio Universal Human Reference RNA (UHRR) dataset, along with a synthetic SIRV (Spike-In RNA Variant) dataset serving as a complex ground truth reference (Fig. 1a).

Performance evaluation was organized into three layers (Fig. 1b): (a) transcript structure analysis, based on precision, recall, and F1-score for isoform reconstruction, novel transcript identification, and TSS/TES detection; (b) isoform quantification, assessed using Pearson and Spearman correlations, CCC, and NRMSE; and (c) computational efficiency, evaluated by runtime and memory usage under identical hardware conditions. This multi-dimensional design supports robust and reproducible comparisons across tools.

SSC classification based on SQANTI [9, 24] revealed that the majority of erroneous events in simulated, SIRV, and real datasets were ISM, NIC, or NNC types (Supplementary Fig. S1). Guided by this, we developed ISAtools, a modular pipeline optimized for reconstructing and quantifying full-length isoforms. The framework comprises six core modules (Fig. 1c, see Materials and methods for details): (1) SSC extraction, (2) SSC preprocessing, (3) SSC polishing, (4) isoform identification, (5) TSS/TES detection, and (6) isoform quantification. ISAtools supports both annotation-guided and de novo modes, and it scales to sequencing depths from 1 M to >1000 M reads.

The SSC analysis pipeline follows a cascaded, multi-stage architecture. The extraction module parses CIGAR strings to define splice junctions and donor-acceptor motifs, outputting both read-level and unique SSC-level records. The preprocessing module filters SSCs by read identity, coverage, and support. The polishing module performs gene-level aggregation, refines junctions using consensus-based correction, and removes fragmented or low-confidence structures. The isoform identification module targets ISM, NIC, and NNC artifacts to preserve high-confidence isoforms. When reference annotations are provided, a dedicated Rescue & Filtering module reintroduces conserved low-abundance SSCs, enhancing isoform completeness.

### Benchmarking isoform identification under diverse annotation conditions

To assess isoform (SSC) identification, we benchmarked ISAtools against IsoQuant, Bambu, Mandalorion, and StringTie [4, 25] across 32 simulated datasets and one SIRV dataset (Fig. 1a). To emulate incomplete annotations, we generated “Reduced Annotation” versions by randomly removing 20% or 40% of transcripts while ensuring one transcript per gene remained, yielding four evaluation scenarios: NoAnno, ReduceAnno20, ReduceAnno40, and FullAnno. A total of 32 simulated datasets and SIRV control data were analyzed, yielding 640 and 20 benchmark instances across annotation settings and methods, respectively.

**Figure 2:**
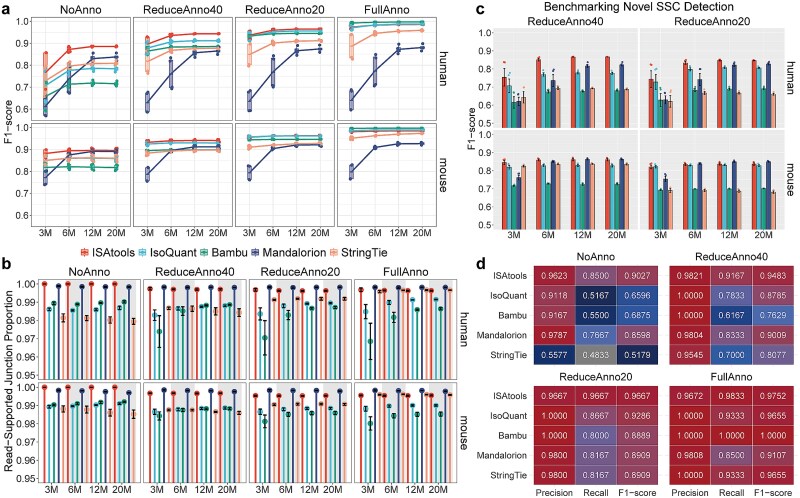
SSC identification accuracy across annotation contexts and transcriptomic complexity; (a) F1-scores for SSC identification across ISAtools, IsoQuant, Bambu, and Mandalorion under FullAnno, ReduceAnno, and NoAnno conditions; (b) read-level support for predicted SSCs, measured as the proportion supported by at least one full-length read; (c) F1-scores for novel SSC identification using 20% and 40% reduced annotations, with ground truth defined by transcripts removed from the reference; (d) SSC-level identification accuracy on the SIRV spike-in dataset across varying annotation completeness.

ISAtools consistently achieved high SSC precision and recall across depths and annotation regimes. Performance gains were most evident under NoAnno conditions, where the absence of reference annotations caused all tools to decline, though ISAtools remained the most stable (Fig. 2a; Supplementary Fig. S2). Human transcriptomes exhibited greater structural complexity than mouse datasets (Supplementary Tables S1 and S2), amplifying performance differentials (Fig. 2a; Supplementary Fig. S2).

To further evaluate performance on low-abundance transcripts, we conducted a stratified sensitivity analysis by grouping isoforms according to their supporting read counts in the ground truth. Recall was computed for each abundance group across different sequencing depths and annotation settings. ISAtools consistently achieved the highest or comparable recall across all abundance levels, with particularly strong performance for ultra-low and low-abundance isoforms (Supplementary Fig. S3). Notably, the performance advantage of ISAtools becomes more pronounced as transcript abundance decreases.

To evaluate read-level support, we examined the fraction of SSCs fully backed by aligned reads. ISAtools and Mandalorion both exceeded 99.5% support across conditions (Fig. 2b), whereas IsoQuant, Bambu, and StringTie exhibited slightly lower rates (96.0%–99.3%, 98.0%–99.2%, and 97.8%–99.4%, respectively).

We next assessed novel isoform discovery by comparing SSC identification in the ReduceAnno20 and ReduceAnno40 conditions. ISAtools outperformed all tools across depths and annotation incompleteness, particularly in human datasets (Fig. 2c; Supplementary Fig. S4). On average, ISAtools improved the F1-score by 4.5% compared to the second-best performing method (IsoQuant, 0.799) across all simulated datasets. This advantage was particularly evident in highly complex transcriptomes with substantially reduced annotations, where ISAtools maintained superior sensitivity for detecting novel isoforms.

In the SIRV dataset (Supplementary Table S3), ISAtools performed on par with IsoQuant and Bambu under FullAnno conditions, but demonstrated superior performance under the ReduceAnno and NoAnno conditions (Fig. 2d), highlighting its robustness in incomplete or absent annotations.

### Benchmarking TSS/TES detection performance

Although long-read sequencing has substantially improved splice junction identification, accurate identification of TSS/TES remains comparatively under explored, despite their critical importance for full-length RNA isoform reconstruction. Recent studies have shown that TSS-TES pairing is globally coordinated and cell-type dependent [21], underscoring the need for flexible, data-driven modeling approaches rather than reliance on static transcript annotations.

**Figure 3:**
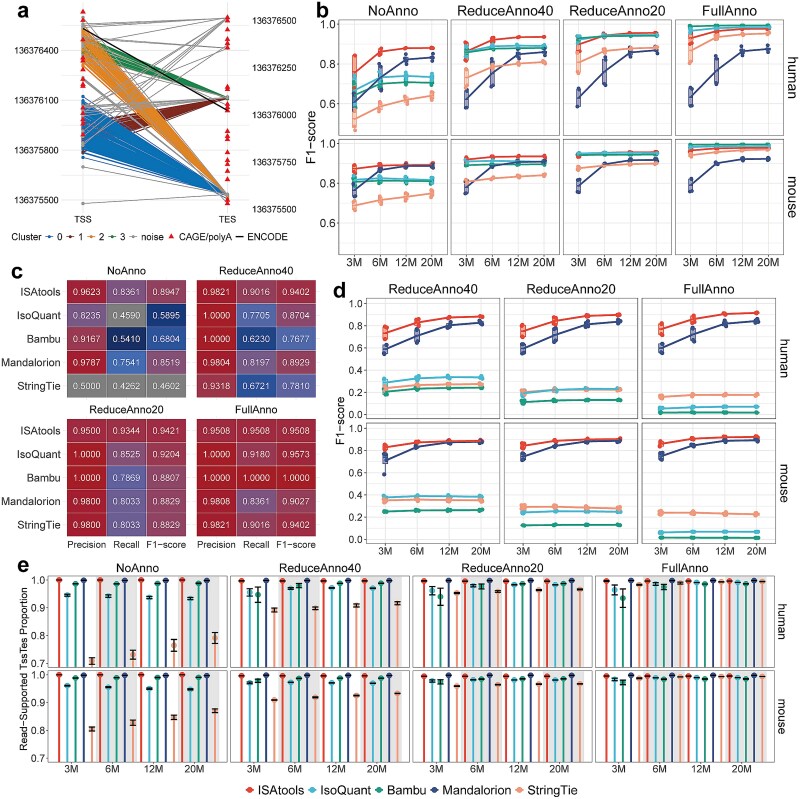
Evaluation of TSS and TES detection across annotation contexts and transcriptomic complexity; (a) schematic of the ISAtools TSS/TES detection strategy; read 5′ and 3′ ends are clustered using a density-based algorithm (DBSCAN) to identify candidate boundaries, followed by read-level reassignment to representative TSS-TES pairs; (b) F1-scores for TSS/TES prediction across ISAtools, IsoQuant, Bambu, Mandalorion, and StringTie under FullAnno, ReduceAnno, and NoAnno conditions in simulated datasets; (c) performance on the SIRV spike-in dataset, reflecting boundary detection robustness in synthetic high-complexity transcriptomes; (d) sensitivity of each tool to boundary annotation, evaluated by comparing prediction accuracy before and after extending reference TSS/TES positions by ±100 bp; (e) fraction of predicted TSS/TES supported by read termini (within ±50 bp), indicating alignment-based evidence.

To address this challenge, ISAtools incorporates a DBSCAN-based clustering module to jointly infer 5′ and 3′ transcript ends from read termini (Fig. 3a). Using the SSC-anchored reads as input, we benchmarked ISAtools against IsoQuant, Bambu, Mandalorion, and StringTie across simulated datasets (640 benchmark instances) and SIRV control data (20 instances) (Fig. 3b–e).

ISAtools consistently delivered high F1-scores across all annotation conditions (FullAnno, ReduceAnno, and NoAnno) (Fig. 3b; Supplementary Fig. S5). The average F1-score reached 0.914 in human datasets and 0.935 in mouse datasets, despite the higher transcriptomic complexity in human (Supplementary Tables S1 and S2). In comparison, the second-best performing method (IsoQuant) achieved lower average F1-scores of 0.880 in human datasets and 0.917 in mouse datasets.

To further assess annotation dependency, we extended reference TSS/TES boundaries by ±100 bp and re-evaluated all methods. ISAtools and Mandalorion maintained stable performance, whereas IsoQuant, Bambu, and StringTie showed marked declines (Fig. 3d), suggesting potential over-reliance on reference boundary definitions. This pattern was further corroborated using the SIRV dataset, in which ISAtools consistently achieved the highest boundary accuracy (Fig. 3c).

We additionally quantified the proportion of predicted TSS/TES sites supported by read termini within ±50 bp. ISAtools and Mandalorion demonstrated robust concordance, exceeding 99.5% across all conditions (Fig. 3e), outperforming IsoQuant (92.9%–99.1%), Bambu (90.3%–99.1%), and StringTie (70.0%–99.5%).

### Benchmarking isoform quantification performance across diverse annotation contexts

Using the 32 simulated datasets generated from SSC-identifiable reads, we systematically evaluated transcript quantification accuracy across five tools: ISAtools, IsoQuant, Bambu, Mandalorion, and Oarfish [26]. We employed multiple metrics-including Pearson correlation, Spearman correlation, CCC, and NRMSE to assess both the accuracy and robustness of quantification across varying transcriptomic complexities and annotation regimes.

**Figure 4:**
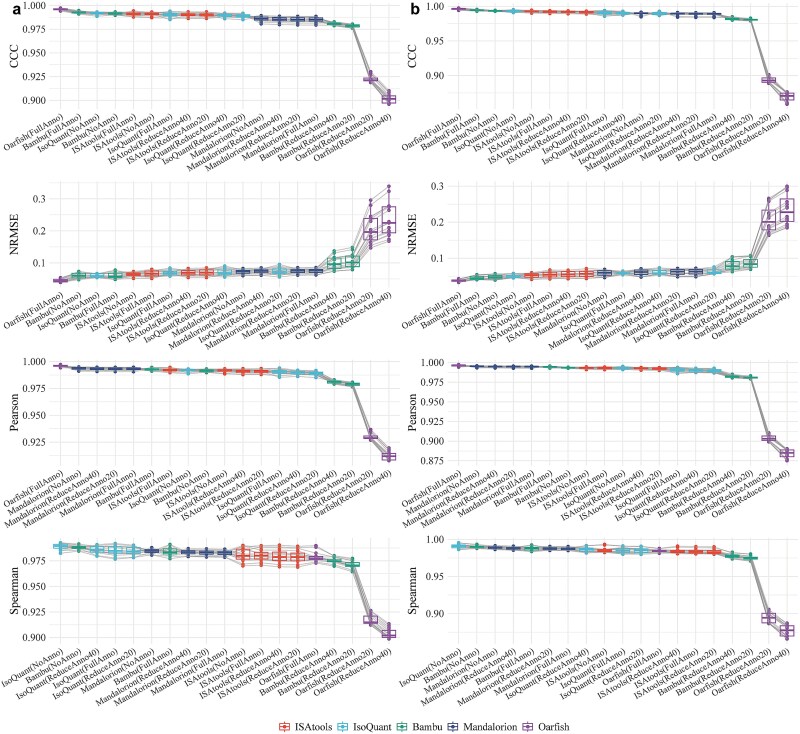
Isoform quantification performance on simulated datasets; quantification accuracy was evaluated using four metrics-CCC, NRMSE, Pearson, and Spearman correlations-across simulated human (a) and mouse (b) datasets (*n* = 32 per species); gray dots represent individual datasets, and gray lines connect results from the same simulated sample under different annotation conditions (FullAnno, ReduceAnno, NoAnno); the central line indicates the median; boxes represent the interquartile range.

As shown in Fig. 4a and b, ISAtools consistently achieved high quantification accuracy across all conditions. On average, ISAtools reached a CCC of 0.991 in human datasets and 0.992 in mouse datasets, with corresponding NRMSE values of 0.067 and 0.055.

IsoQuant showed comparable performance, with slightly lower CCC (0.990 / 0.991) and marginally higher NRMSE (0.068/0.058) in human and mouse datasets, respectively. Mandalorion achieved high Pearson correlations (0.993–0.995) but lower CCC values. Bambu showed greater variability, although it achieved high CCC and low NRMSE under both FullAnno and NoAnno conditions.

Oarfish, a dedicated quantification method based on a coverage-aware probabilistic model for read assignment, achieved the highest accuracy under FullAnno conditions (CCC: 0.997; Pearson: 0.997; NRMSE: 0.032), but showed substantially reduced performance under incomplete annotation settings.

In correlation analysis, Mandalorion achieved the highest Pearson correlation, while Spearman correlations were comparable across methods, with ISAtools and IsoQuant showing similar performance. Bambu and Oarfish exhibited greater variability, particularly under incomplete annotations.

### Isoform reconstruction performance on real biological samples

To assess ISAtools under realistic conditions, we benchmarked performance on the PacBio UHRR dataset, which includes six technical replicates sequenced using the Kinnex full-length RNA-seq protocol and Revio SPRQ chemistry, producing ~80 million high-quality cDNA reads. Given the absence of ground-truth isoforms, we evaluated concordance with GENCODE annotations, boundary-level accuracy, and empirical read support, excluding any isoforms with an estimated count of fewer than three supporting reads.

**Figure 5:**
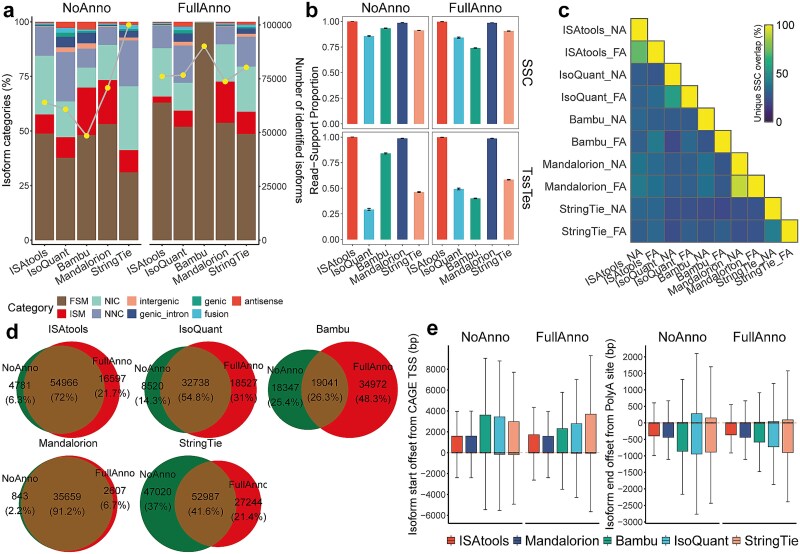
Performance of isoform reconstruction and boundary detection on real long-read RNA-seq datasets; (a) isoform classification results from SQANTI3 using GENCODE v38 as reference, under FullAnno and NoAnno conditions; bar plots show the proportion of each SQANTI category; overlaid lines indicate the total number of predicted isoforms; (b) read-level support for predicted isoforms, assessed at two levels: SSC support, defined as at least one full-length read matching the SSC; and SSC + ends support (TSS/TES level), which additionally requires read termini to fall within ±50 bp of the predicted transcript start and end sites; (c) overlap of unique SSCs predicted under FullAnno (FA) and NoAnno (NA) conditions; (d) distribution of unique SSCs detected exclusively under FullAnno or NoAnno conditions; (e) distribution of distances between predicted TSS/TES and experimentally defined sites from refTSS v3.1 and polyASite v2.0.

First, we performed a comparative analysis of isoforms detected by each tool against the GENCODE annotation using SQANTI3 [24]. Under the NoAnno condition, ISAtools detected a mean of 68 721 isoforms per sample, with 49.3% classified as full splice match (FSM). Mandalorion recovered a similar number (70 359), with a higher FSM proportion (53.2%). IsoQuant detected fewer isoforms (60 200) and a lower FSM rate (37.7%), while Bambu produced the fewest isoforms (47 364) with an FSM rate of 48.7%. In contrast, StringTie reported a substantially higher number of isoforms (100 007) but with a relatively low FSM proportion (30.0%) (Fig. 5a; Supplementary Fig. S6).

Under the FullAnno condition, ISAtools identified 74 961 isoforms, of which 63.1% were classified as FSMs. Mandalorion reported 73 251 isoforms with an FSM proportion of 53.9%, while IsoQuant detected 75 766 isoforms with 51.8% classified as FSMs. Although Bambu identified a relatively large number of isoforms (88 961), it exhibited strong dependence on reference annotations, with 99.6% of its predictions classified as FSMs, indicating minimal identification of novel isoforms. StringTie reported 80 293 isoforms, with 48.7% classified as FSMs. Notably, this evaluation was performed on the UHRR dataset, which represents a highly complex transcriptomic sample derived from mixed RNA sources.

We next evaluated empirical support using two criteria: (i) SSC support, defined as having at least one read perfectly matching the isoform’s SSC, and (ii) SSC + ends (SSC + E) support, requiring in addition that read alignment start and end sites be within 50 nt of the isoform’s transcript boundaries. ISAtools and Mandalorion maintained >99.7% and >98.6% SSC + E support, respectively. IsoQuant showed 83.4%–86.6% SSC support, but only 27.4%–50.3% SSC + E support. Bambu’s SSC + E support varied widely (39.6%–84.7%), with a paradoxical drop upon adding reference annotation, indicating potential boundary overfitting. StringTie achieved high SSC support (~91% in both conditions), while SSC + E support was comparatively lower (~46% in NoAnno and ~58% in FullAnno) (Fig. 5b).

We also measured SSC consistency between NoAnno and FullAnno conditions. Mandalorion showed the highest cross-annotation consistency (~91%). ISAtools and IsoQuant followed (~72% and ~55%), while StringTie and Bambu exhibited lower overlap (~42% and ~ 26%, respectively) (Fig. 5c; Supplementary Fig. S7).

Further analysis revealed distinct identification patterns (Fig. 5d; Supplementary Fig. S8). ISAtools showed balanced annotation-specific sensitivity, with 6.3% and 21.7% of unique SSCs exclusive to NoAnno and FullAnno, respectively, reflecting adaptability to both novel and reference-supported isoforms. IsoQuant followed a similar trend, though skewed more toward annotation reliance (14.3% NoAnno-exclusive; 31% FullAnno-exclusive).

StringTie showed a higher proportion of annotation-specific isoforms, with 37.0% and 21.4% of unique SSCs identified exclusively under NoAnno and FullAnno conditions, respectively. Bambu exhibited the highest annotation sensitivity, with 25.4% and 48.3% of unique SSCs identified exclusively under NoAnno and FullAnno, respectively.

In contrast, Mandalorion, consistent with its conservative identification strategy, showed the lowest proportion of annotation-exclusive SSCs (2.2% NoAnno-exclusive; 6.7% FullAnno-exclusive), indicating minimal dependence on reference annotations but also reduced sensitivity to novel isoforms.

For TSS/TES validation, predicted transcript start and end sites were compared against known sites from the Reference Transcription Starting Sites [27] (refTSS, v3.1) database and polyASite [28] database (v2.0). The distance from each predicted TSS/TES to its nearest reference site was calculated and summarized as boxplots (Fig. 5e). ISAtools and Mandalorion showed similar distributions for TSS, with distances tightly clustered near the reference, indicating precise start site recovery. For TES, ISAtools predictions were more concentrated than those of other tools, reflecting higher boundary precision and better concordance with polyadenylation sites.

### Comparison of computational efficiency

We compared the runtime and memory usage of ISAtools, IsoQuant, Bambu, Mandalorion, and StringTie across all simulated datasets on the same computational platform (see Materials and methods). Since Mandalorion operates on unaligned reads, only its PDFQ module was evaluated for fair comparison.

**Figure 6:**
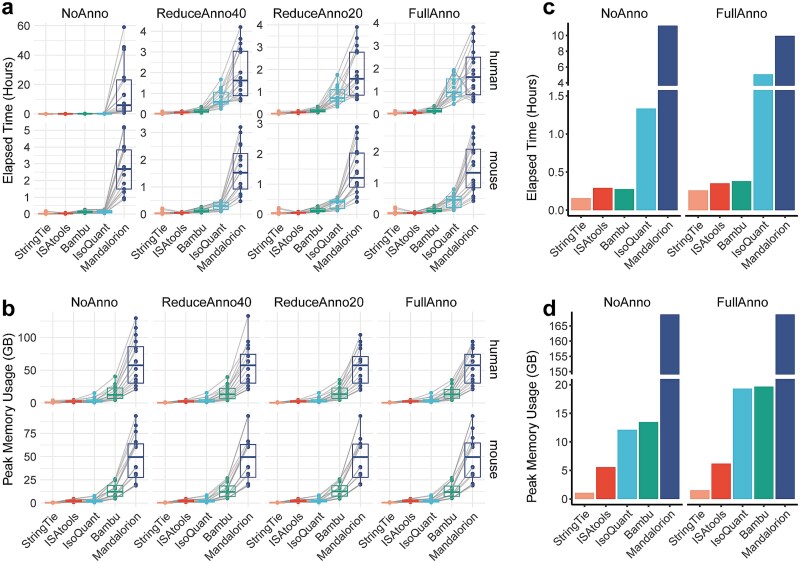
Computational efficiency of isoform reconstruction tools; (a-b) runtime and peak memory usage across 32 simulated datasets under different annotation conditions. Mandalorion was evaluated using only its PDFQ module to ensure comparability; (c-d) runtime and memory usage for multi-sample analysis of the UHRR dataset (~80 million cDNA reads).

As shown in Fig. 6a and b, StringTie consistently achieved the fastest runtime and lowest memory usage across all conditions (average ~ 2.5 min, ~0.6 GB). ISAtools showed the next best performance (~3.5 min, ~2.5 GB). Bambu generally outperformed IsoQuant, while IsoQuant exhibited increased runtime under FullAnno due to annotation database construction overhead. Mandalorion incurred the highest computational cost across all settings.

On UHRR datasets (~80 million reads), StringTie completed the analysis in ~10–16 min with low memory usage (~1–1.6 GB), while ISAtools required ~17–21 min with moderate memory usage (~5–6 GB) (Fig. 6c and d). Disk I/O evaluation revealed that Bambu incurred the lowest read/write demands, whereas Mandalorion’s disk usage was ~30-fold higher, limiting its scalability (Supplementary Figs S9 and S10).

To assess scalability on large-scale datasets, we simulated 50 samples comprising ~1 billion reads in total. ISAtools completed isoform reconstruction and quantification within ~4 h using ~8 GB of memory. StringTie required longer runtime (~6 h) but maintained a lower memory footprint (~1.5 GB). In contrast, IsoQuant, Bambu, and Mandalorion failed to complete the analysis due to excessive memory requirements (Table 1). These results highlight the scalability of ISAtools for billion-read-scale long-read RNA-seq datasets.

**Table 1:** Scalability and resource usage of isoform analysis tools at billion-read scale under FullAnno condition.

Tool	Dataset size	Completed	Elapsed time (h)	Maximum resident set size (GB)
ISAtools	~1B reads	Yes	~4 h	~8 GB
StringTie	~1B reads	Yes	~6 h	~1.5 GB
IsoQuant	~1B reads	No	–	>300 GB^a^
Bambu	~1B reads	No	–	>300 GB^a^
Mandalorion	~1B reads	No	–	>300 GB^a^

^a^IsoQuant, Bambu, and Mandalorion were run with a 300 GB memory limit but did not complete due to memory exhaustion.

–, not available.

## Discussion

The rapid adoption of the PacBio Revio platform, which combines high throughput with high-fidelity long-read sequencing, has created an urgent demand for specialized computational methods capable of efficiently processing hundreds of millions of reads while maintaining accurate full-length isoform reconstruction. ISAtools directly addresses this need through a set of interconnected algorithmic innovations specifically optimized for the characteristics of PacBio CCS data.

The central methodological advance of ISAtools is the introduction of the SSC representation, which transforms complex splice graphs into standardized, text-based strings that support efficient set operations and large-scale parallelization. This design fundamentally differs from the graph-centric strategies employed by tools such as IsoQuant and related methods, yielding substantial performance gains while preserving analytical rigor. The SSC framework functions as a unifying data structure that seamlessly links read alignments, reference annotations, and downstream computational modules, thereby enabling both annotation-guided and de novo isoform reconstruction within a consistent analytical framework.

**Figure 7:**
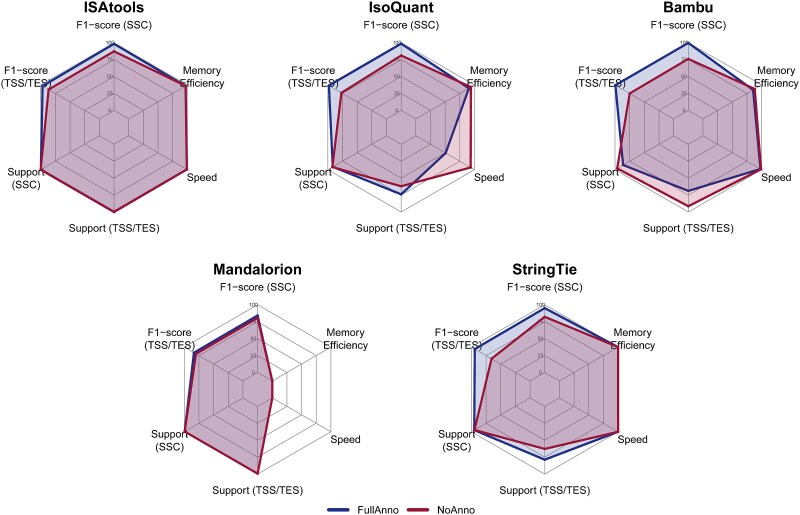
Comprehensive assessment across evaluation dimensions; overall performance was evaluated across six key metrics under both FullAnno and NoAnno conditions: F1-score for SSC and TSS/TES detection, read-level support for SSCs and transcript boundaries, computational speed, and memory efficiency; F1-scores were averaged across simulated datasets per annotation condition; support scores were computed as the mean of simulated and real datasets; speed and memory metrics were averaged across datasets and normalized across tools; ISAtools shows balanced and robust performance across all dimensions.

Comprehensive benchmarking demonstrates that ISAtools achieves superior and stable performance across multiple evaluation dimensions (Fig. 7). Notably, under no-annotation conditions—a critical scenario for non-model organisms and exploratory biological contexts—ISAtools retains ~95% of its full-annotation performance, substantially outperforming existing approaches. This robustness arises from its data-driven architecture, which prioritizes read-level evidence over annotation priors while incorporating targeted error correction for systematic artifacts characteristic of PacBio CCS data.

Accurate delineation of transcript boundaries remains a critical yet under-addressed challenge in long-read transcriptomics. ISAtools addresses this through an unsupervised, data-driven approach to transcription start and end site inference based on read termini distributions. By avoiding explicit reliance on reference annotations, this strategy captures sample-specific transcriptional architecture, including alternative promoter usage and variable polyadenylation, which are frequently underestimated by annotation-dependent methods. The resulting improvement in boundary precision is particularly relevant for studies focused on isoform regulation rather than catalog completeness.

Beyond accuracy, ISAtools delivers substantial gains in computational efficiency and scalability. In real PacBio Revio datasets comprising tens of millions of reads, ISAtools completes isoform reconstruction within minutes while maintaining a modest memory footprint. More importantly, it scales to billion-read datasets**—**corresponding to dozens of deeply sequenced samples**—**using <10 GB of memory and completing analysis within a few hours. In contrast, alternative tools were unable to complete these large-scale analyses even when allocated substantially higher memory resources. This level of efficiency transforms billion-read Iso-Seq analysis from a specialized, resource-intensive task into a practical component of routine transcriptomic workflows.

Several limitations merit consideration. First, ISAtools is currently optimized for PacBio CCS data. Direct application to ONT sequencing is not supported with the current version; handling ONT data will require code updates and extension of the framework with adaptive error modeling to accommodate higher and more heterogeneous error rates. We are actively working on extending ISAtools to ONT datasets in future versions [29]. Second, the conservative filtering strategy used to suppress false-positive isoforms may reduce sensitivity for extremely low-abundance transcripts. While this trade-off is appropriate for most bulk and high-depth datasets, studies focused on rare isoforms or single-cell long-read applications may benefit from parameter tuning. Finally, genomic regions with extreme splicing complexity, dense repeats, or high sequence homology remain challenging for all isoform reconstruction methods, including ISAtools, and may require integration with orthogonal experimental evidence.

Beyond its immediate applications, the SSC framework underlying ISAtools has broader methodological implications for transcriptomics. Its text-based representation enables efficient isoform comparison across samples, tissues, and species using simple string operations, facilitating large-scale comparative transcriptomic analyses. This paradigm may be particularly advantageous for emerging applications such as single-cell long-read RNA sequencing, where sparse coverage necessitates highly efficient data structures, or for integrative analyses combining transcriptomic and epigenomic information to infer isoform-specific regulatory functions.

Looking forward, long-read RNA sequencing is entering a phase in which improvements in sequencing accuracy are increasingly matched by dramatic gains in throughput. As these trends continue, computational efficiency will become a dominant constraint on biological discovery. In this context, scalable isoform reconstruction strategies built on efficient representations**—**such as the SSC framework**—**are likely to play a defining role, analogous to the impact of highly optimized short-read alignment and assembly frameworks, including StringTie [25] and HISAT [30], during the maturation of short-read RNA sequencing.

In conclusion, ISAtools provides a specialized, efficient, and accurate solution for full-length RNA isoform reconstruction from PacBio CCS data. Its balanced performance across annotation conditions, robust transcript boundary detection, and exceptional computational efficiency make it particularly well-suited for the growing number of studies leveraging high-throughput PacBio sequencing. By reducing dependence on static reference annotations while maintaining biological fidelity, ISAtools enables high-resolution transcriptome profiling across diverse biological systems and empowers researchers to fully realize the potential of modern long-read RNA sequencing technologies.

**Key Points**

- ISAtools introduces a splice site chain representation that enables efficient integration of read alignments and transcript annotations for large-scale long-read isoform analysis.
- ISAtools accurately recovers both annotated and novel full-length isoforms, retaining ~95% of its performance even in the absence of reference annotations.
- ISAtools applies unsupervised, data-driven inference of transcription start and end sites, enabling precise transcript boundary delineation without annotation dependence.
- ISAtools achieves high computational efficiency, scaling to nearly 1 billion PacBio circular consensus sequencing reads within a few hours while using <10 GB of memory.

## Supplementary Material

Supplementary_file_bbag403

## Data Availability

All datasets used in this study are publicly available. PacBio long-read sequencing data from *Mus musculus* cortex and heart tissues, used for IsoSeqSim simulations, were obtained from the ENCODE database under file IDs: ENCFF565RLW, ENCFF325BXV, ENCFF584WWA, and ENCFF860CBL. Human prefrontal cortex and left/right ventricular heart tissue data were also obtained from ENCODE under file IDs: ENCFF708BOP, ENCFF827DUW, ENCFF537NCV, and ENCFF615FIC. The SIRV dataset was derived from the SIRV subset of the PacBio Iso-Seq UHRR collection, available at https://downloads.pacbcloud.com/public/dataset/UHR_IsoSeq/. The high-depth PacBio UHRR dataset (Revio platform, SPRQ chemistry) is available at https://downloads.pacbcloud.com/public/dataset/Kinnex-full-length-RNA/DATA-RevioSPRQ-UHRR2024/. Source data for all figures are provided as Supplementary Data.

## Code availability

ISAtools is available at: https://github.com/Chenhu7/ISAtools.
