# Supplementary material for: Efficient reconstruction of full-length RNA isoforms using ISAtools and large-scale PacBio circular consensus sequencing data
Source: Brief Bioinform. 2026 Jul 30;27(4):bbag403. doi: 10.1093/bib/bbag403 (PMC13435227; doi:10.1093/bib/bbag403)
Supplement: Supplementary file 1 [file supplementary_file_bbag403.pdf]

Supplementary Information

TABLE OF CONTENTS

Supplementary Figures

Supplementary Figure 1. SQANTI Classification of SSCs Across Datasets.

Supplementary Figure 2. Precision and Recall of SSC Identification.

Supplementary Figure 3. Sensitivity Analysis Across Transcript Abundance Gradients.

Supplementary Figure 4. Precision and Recall of novel SSC Detection.

Supplementary Figure 5. Precision and Recall of TSS/TES Identification.

Supplementary Figure 6. SQANTI Classification of Isoforms Identified by Different Tools.

Supplementary Figure 7. Similarity of Predicted Unique SSC.

Supplementary Figure 8. Overlap of Unique SSCs Identified Under No and Full Annotation per Tool.

Supplementary Figure 9. Distribution of Disk I/O Across Simulated Datasets.

Supplementary Figure 10. Distribution of Disk I/O Across UHRR.

Supplementary Figure 11. Methodological Comparison Between ISAtools_evaluation and Gffcompare

Supplementary Tables

Supplementary Table 1. Simulated Data (human) Summary Based on Real Transcriptomic Profiles for Benchmarking.

Supplementary Table 2. Simulated Data (mouse) Summary Based on Real Transcriptomic Profiles for Benchmarking.

Supplementary Table 3. Transcriptomic Complexity of UHRR and SIRV Datasets.

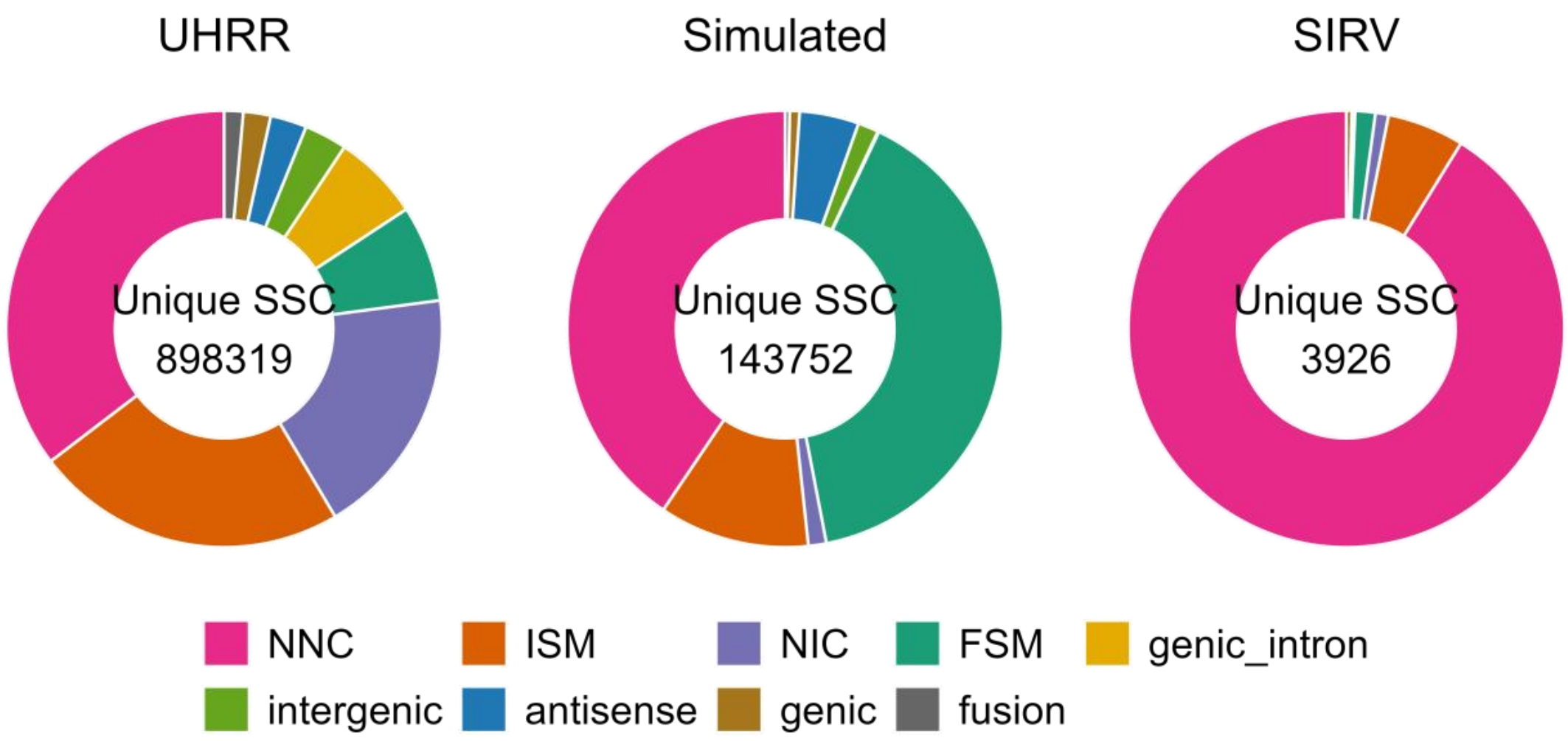

Supplementary Figure 1 | SQANTI classification of SSCs across datasets. Based on real RNA-seq data, simulated, and SIRV data, we analyzed SSCs using SQANTI. Most errors fall into the ISM (Incomplete Splice Match), NIC (Novel In Catalog), and NNC (Novel Not in Catalog) categories, while FSM (Full Splice Match) represents accurate matches to known annotations.

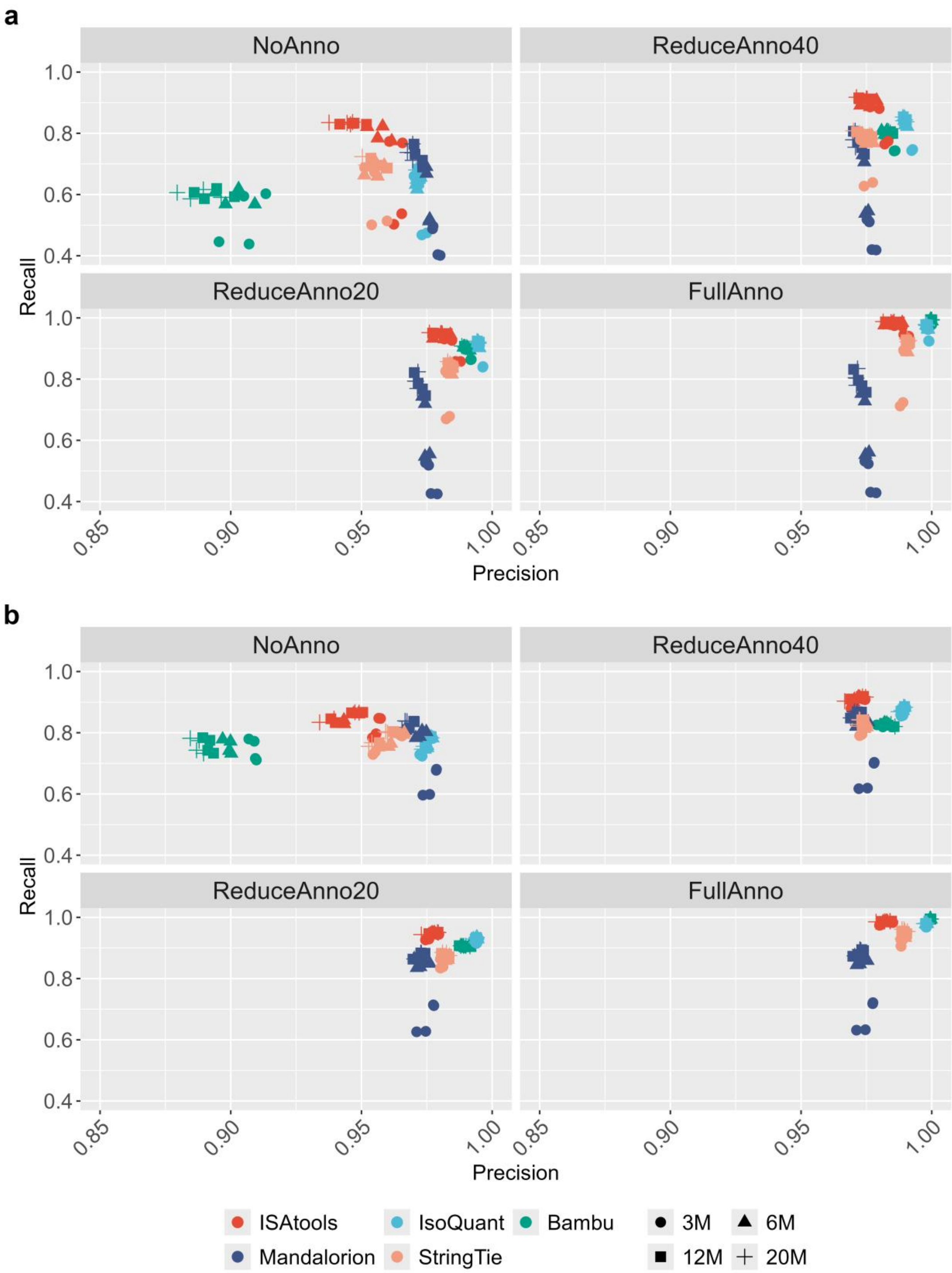

Supplementary Figure 2 | Precision and recall of SSC identification. Each point represents precision and recall for SSC identification at varying sequencing depths and annotation conditions in simulated human (**a**) and mouse (**b**) datasets.

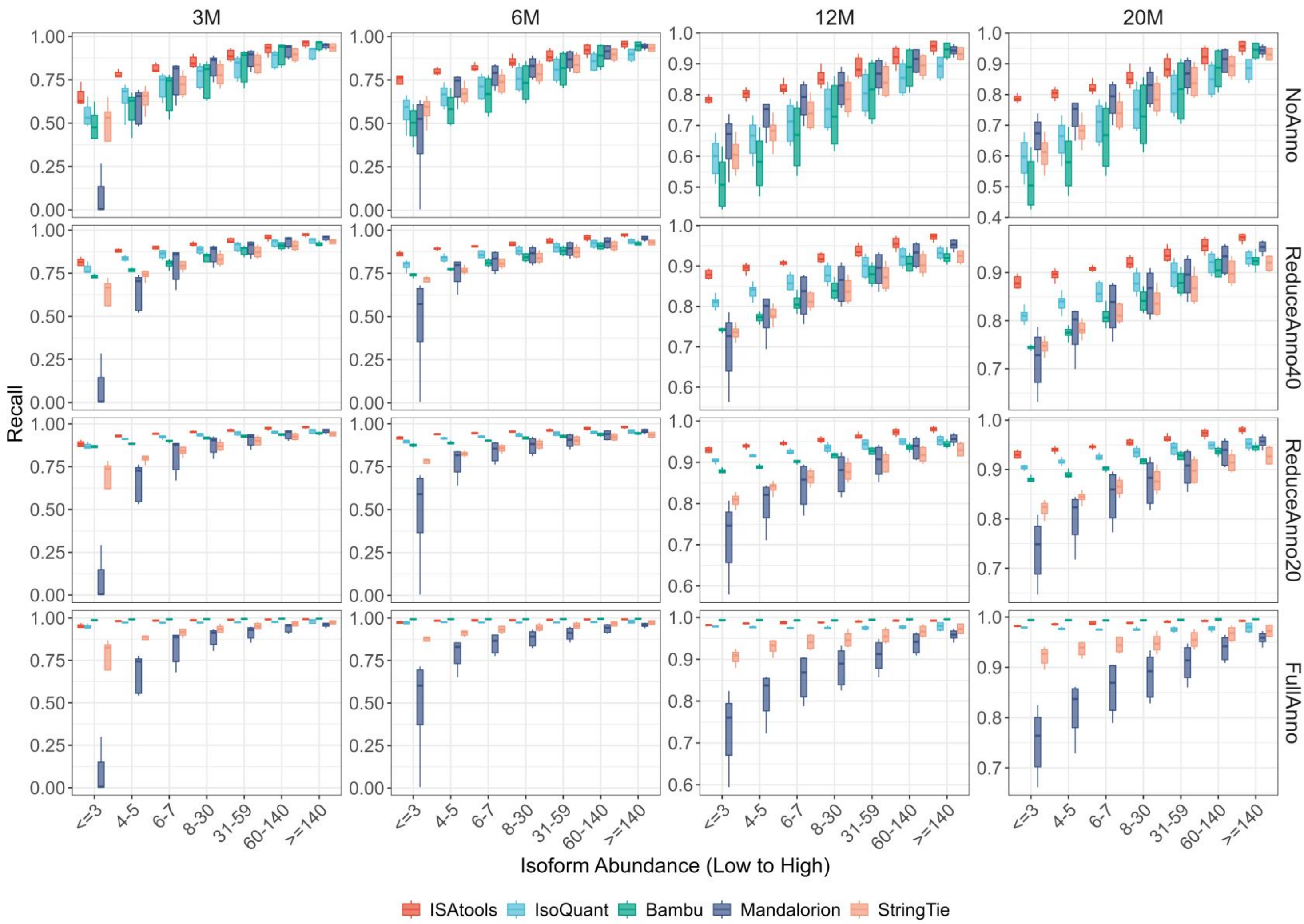

Supplementary Figure 3 | Sensitivity analysis across transcript abundance gradients. Recall performance of ISAtools and competing methods stratified by isoform abundance. Ground-truth isoforms were grouped into bins based on supporting read counts (from low to high abundance). Rows represent different annotation settings, and columns correspond to increasing sequencing depths. Boxplots show the distribution of recall across replicates.

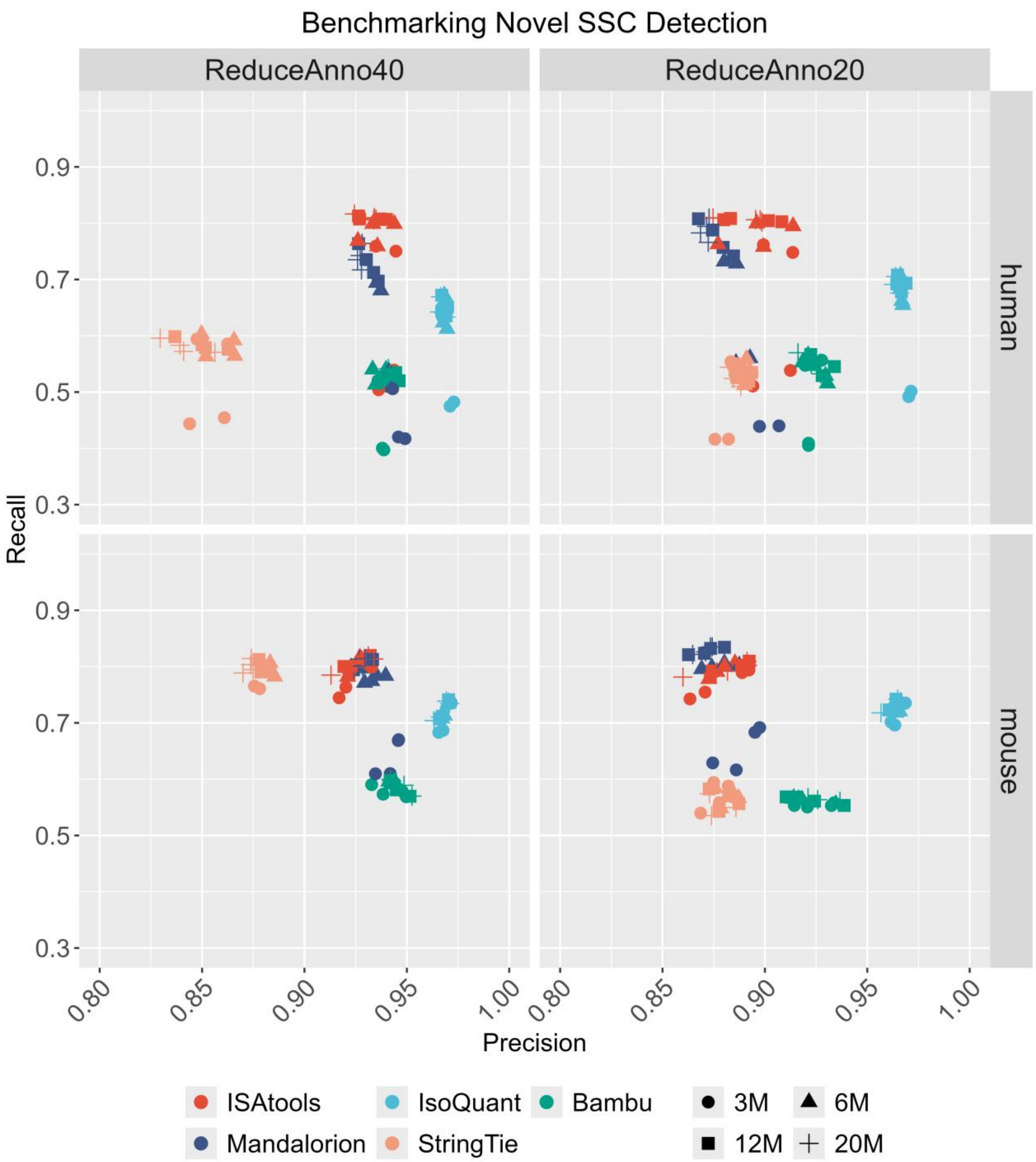

Supplementary Figure 4 | Precision and recall of novel SSC discovery. Each point represents the precision and recall of novel SSC detection at varying sequencing depths under reduced annotation in simulated human and mouse datasets.

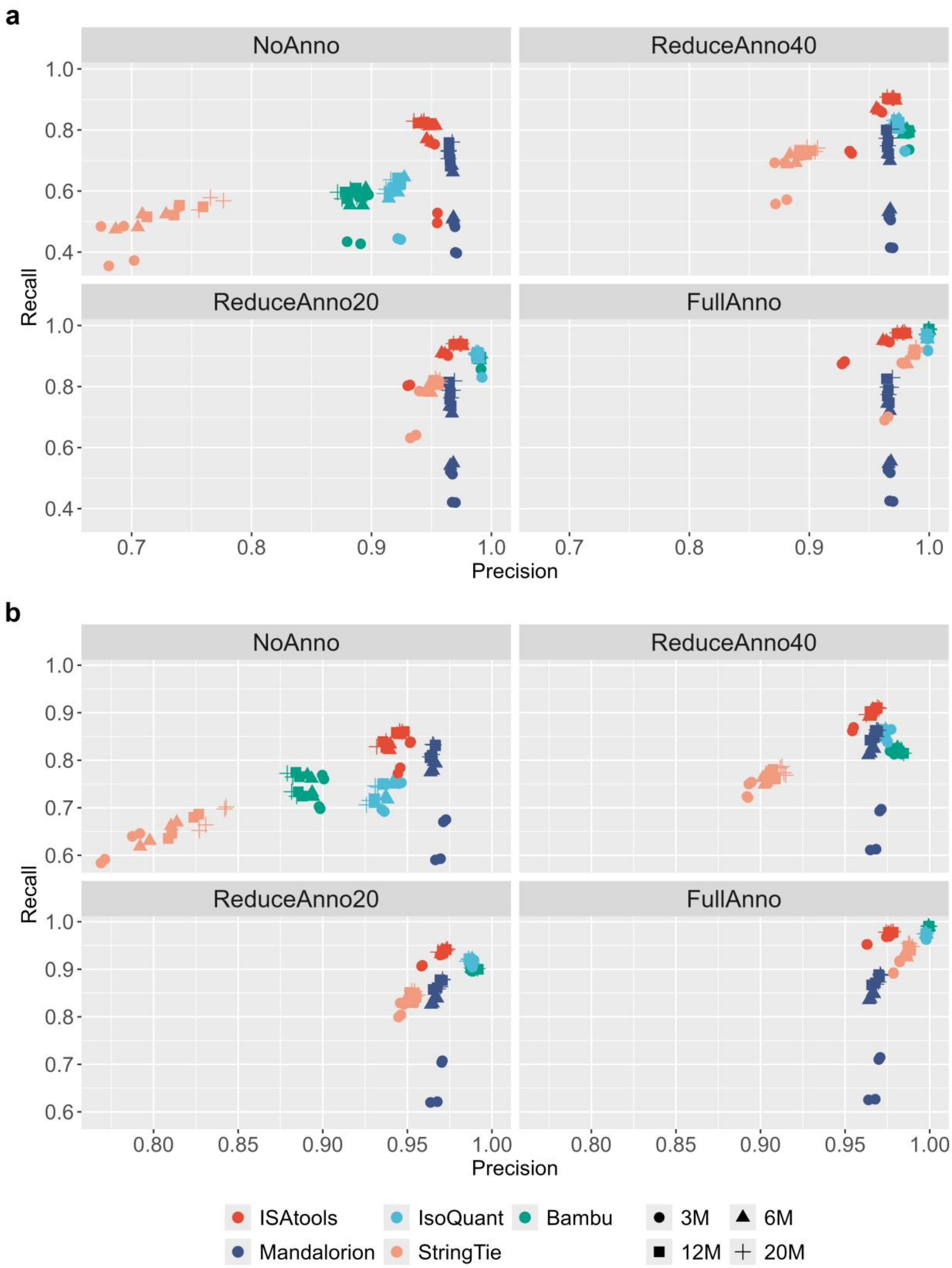

Supplementary Figure 5 | Precision and Recall of TSS/TES Detection. Each point represents precision and recall for TSS/TES detection at varying sequencing depths and annotation conditions in simulated human (**a**) and mouse (**b**) datasets.

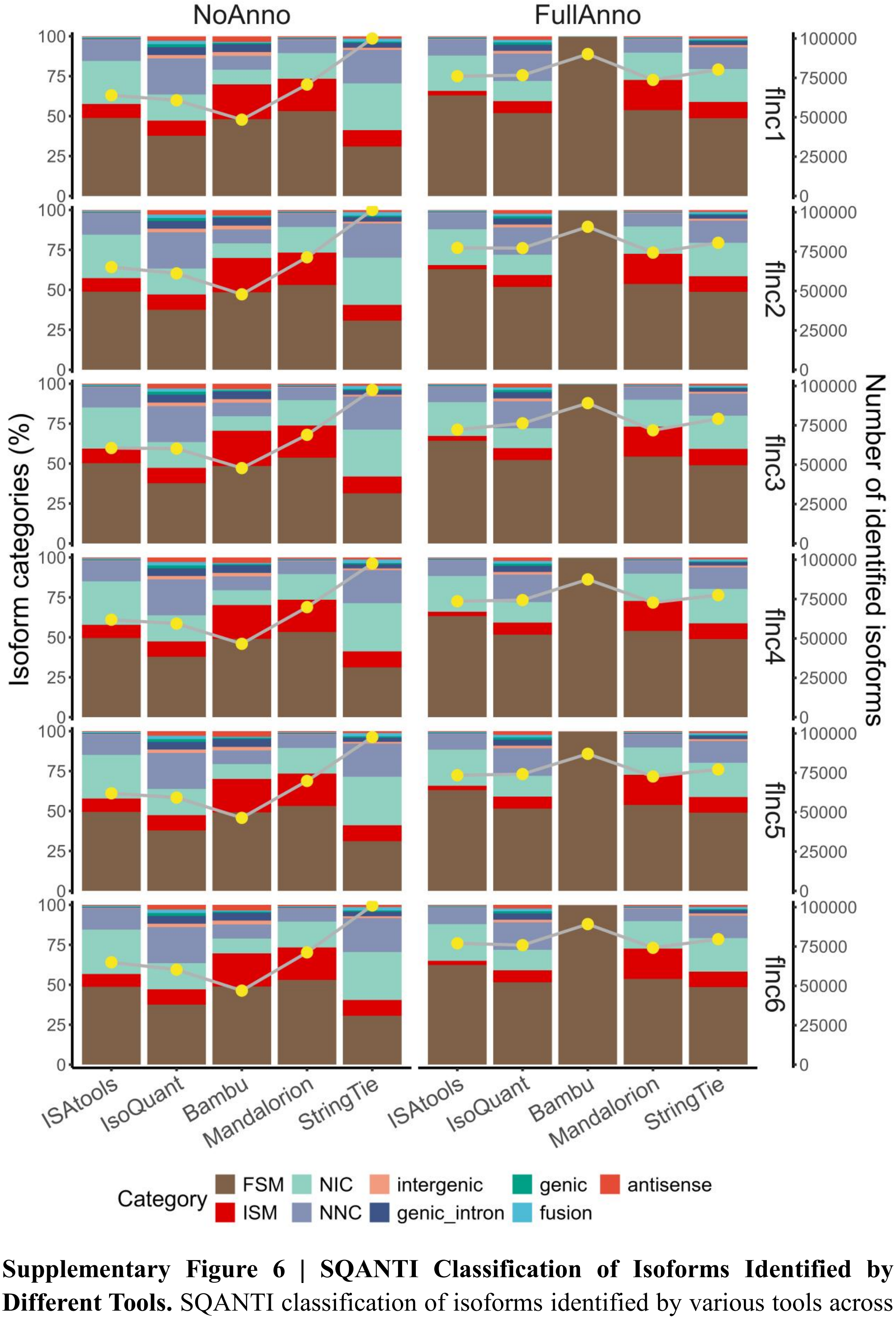

Supplementary Figure 6 | SQANTI Classification of Isoforms Identified by Different Tools. SQANTI classification of isoforms identified by various tools across six UHRR samples (flnc1-6). For each sample, bar plots (left y-axis) show the percentage of isoforms in each SQANTI category, while line plots (right y-axis) indicate the total number of predicted isoforms.

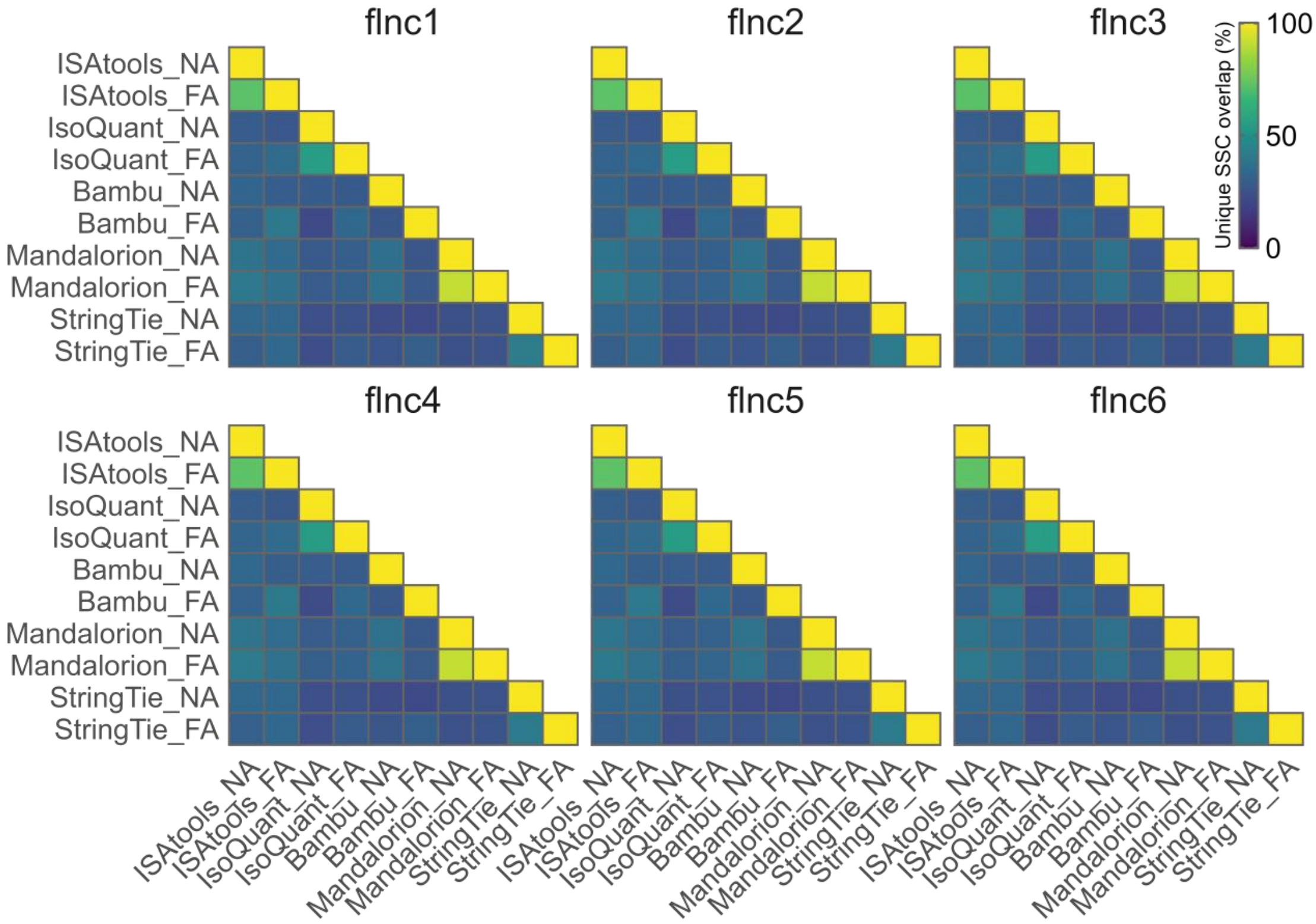

Supplementary Figure 7 | Similarity of Predicted Unique SSC. Heatmap showing pairwise similarity of unique SSCs predicted by different tools under full annotation (FA) and no annotation (NA) conditions.

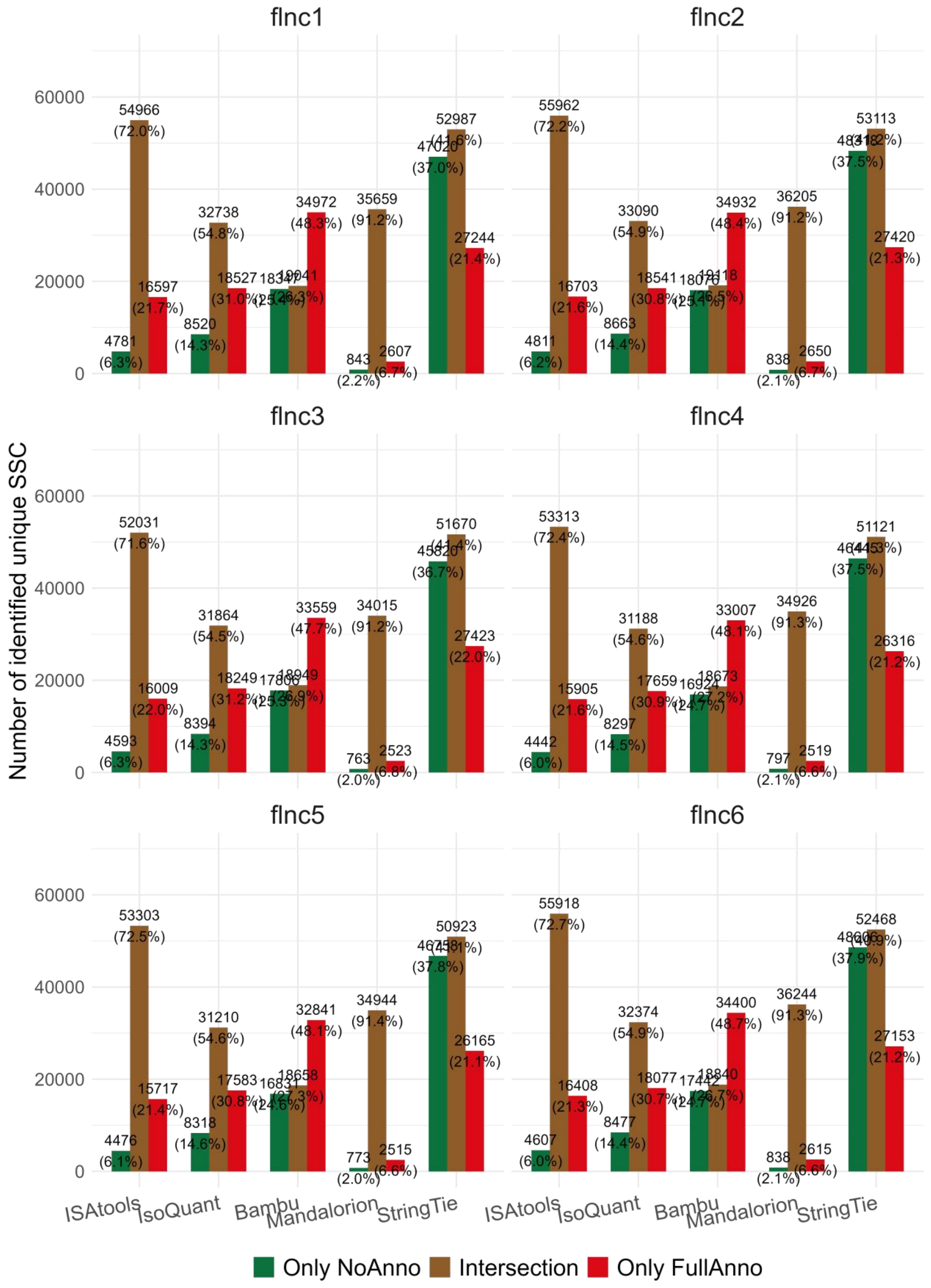

Supplementary Figure 8 | Overlap of Unique SSCs Identified Under No and Full Annotation per Tool. Bar plots showing the number of unique SSCs identified by each tool under no annotation only (left bar), both annotation conditions (middle bar, intersection), and full annotation only (right bar), across six UHRR samples. The distribution illustrates each tool's sensitivity to the presence or absence of reference annotation.

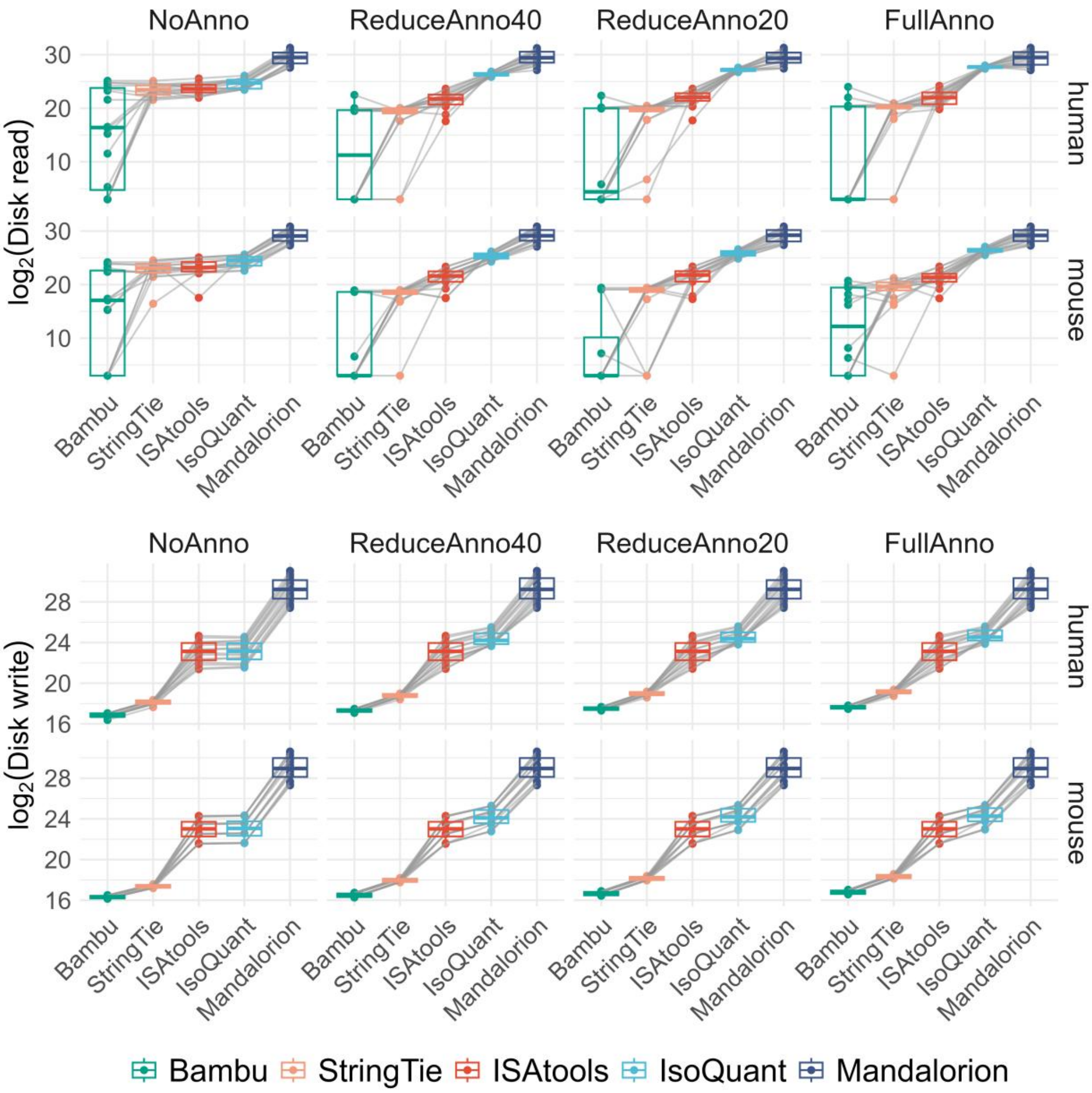

Supplementary Figure 9 | Distribution of Disk I/O Across Simulated Datasets. Boxplots showing the distribution of disk read (a) and write (b) volumes across all simulated datasets. Each dot represents an individual measurement, and lines connect values from the same simulation, illustrating paired relationships. The y-axis represents the $\log_2$-transformed number of file system input/output blocks (where 1 block = 512 bytes), reflecting the cumulative data volume transferred to/from the storage device, as reported by the File system inputs and File system outputs fields of `/usr/bin/time -v`.

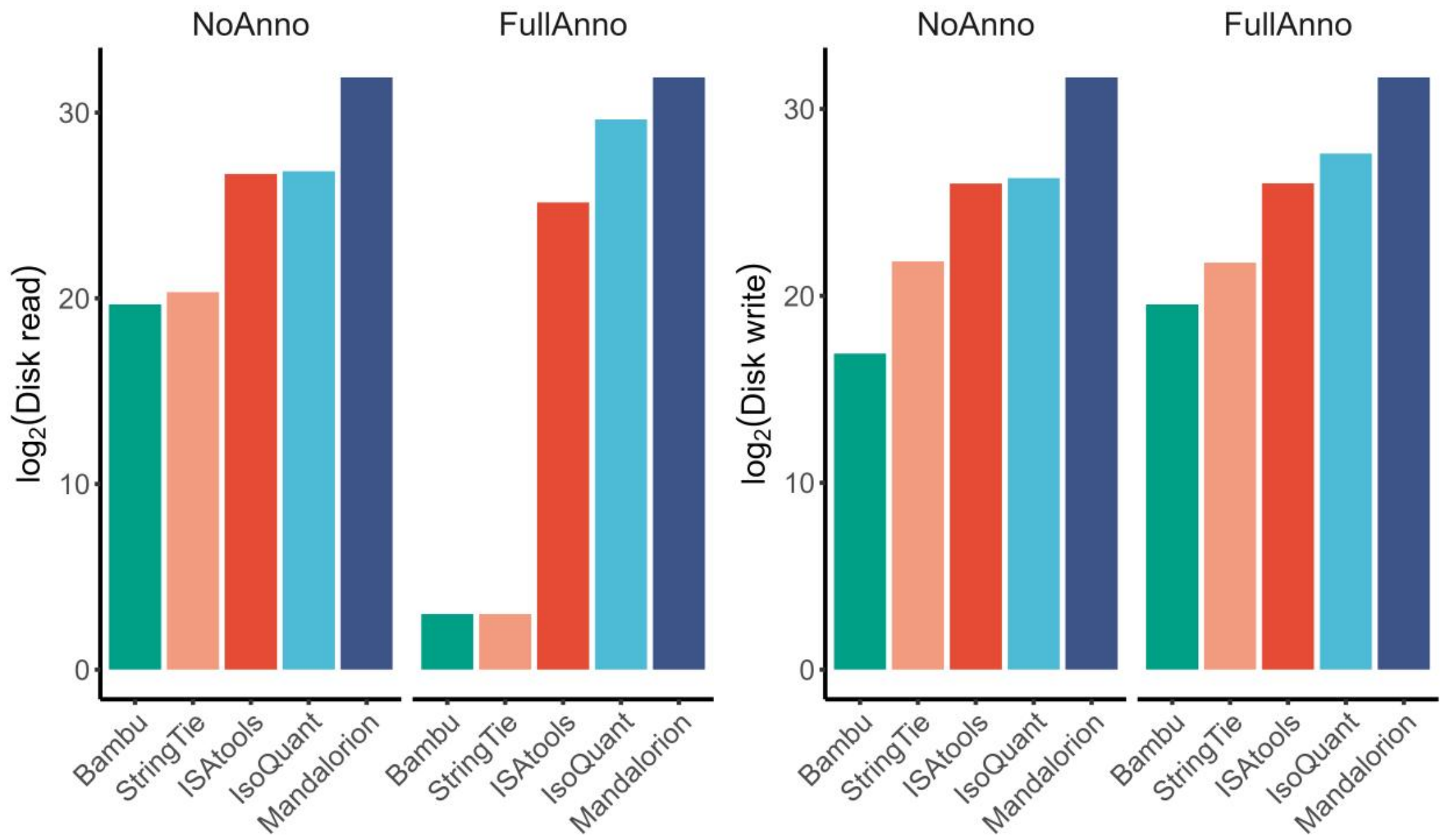

Supplementary Figure 10 | Distribution of Disk I/O Across UHRR. Bar plots showing the total disk read (a) and write (b) volumes for each tool on the full UHRR dataset under multi-sample mode. The y-axis represents the $\log_2$-transformed number of file system input/output blocks (where 1 block = 512 bytes), reflecting the cumulative data volume transferred to/from the storage device, as reported by the File system inputs and File system outputs fields of `/usr/bin/time -v`.

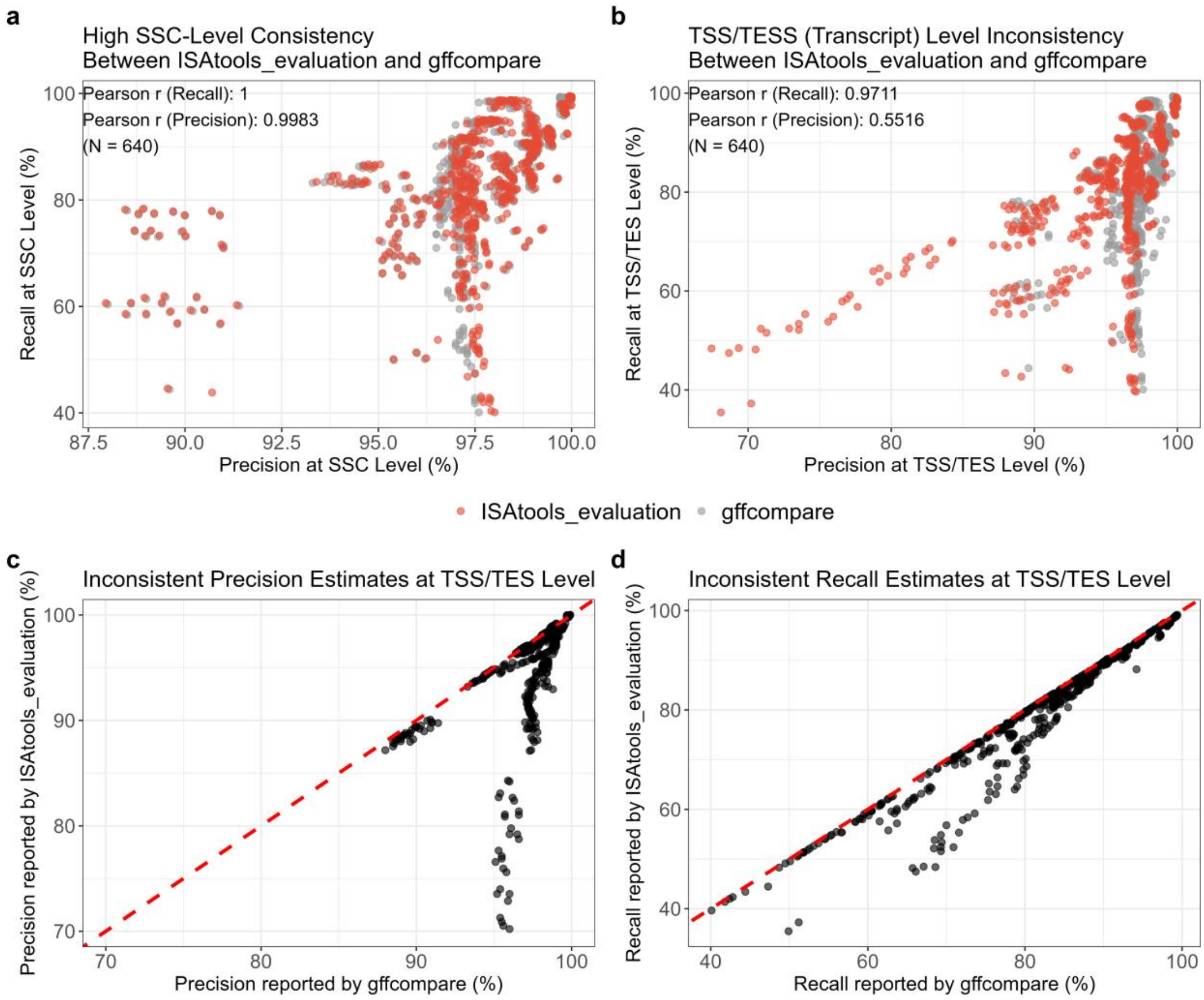

Supplementary Figure 11 | Methodological comparison between ISAtools_evaluation and gffcompare. a-b, Comparison of precision and recall at the SSC level (**a**) and TSS/TES level (**b**) between ISAtools_evaluation (red) and gffcompare (grey). Each dot represents a dataset or simulation condition. Results are highly consistent at the SSC level, whereas differences are observed for TSS/TES evaluation. **c-d**, Correlation of precision (**c**) and recall (**d**) estimates between the two frameworks for TSS/TES evaluation.

Supplementary Tables

			Simulated GTF (Transcript Annotation)			Simulated Sequencing Data (Reads)		
**Species**	**Biosample summary**	**Accession**	**Total Genes**	**Total Isoforms**	**Isoforms per Gene**	**Simulated Depth (Read Count)**	**Total Unique SSC**	**Average Read Support per SSC**
Human	Dorsolateral prefrontal cortex	ENCFF708BOP	24025	83505	3.5	3M	110705	26
						6M	131130	43
						12M	160137	71
						20M	188715	100
		ENCFF827DUW	24089	80748	3.4	3M	115535	25
						6M	136968	42
						12M	168245	68
						20M	201586	94
	Heart left ventricle	ENCFF537NCV	17485	58295	3.3	3M	89718	33
						6M	111355	53
						12M	143752	82
						20M	176776	111
		ENCFF615FIC	18600	59805	3.2	3M	101604	29
						6M	127363	46
						12M	166524	70
						20M	209897	93

Supplementary Table 1 | Simulated Data (human) Summary Based on Real Transcriptomic Profiles for Benchmarking. This table presents simulated datasets generated by IsoSeqSim using expression profiles derived from real transcriptomic data. It includes transcript annotation complexity (e.g., gene and isoform counts, isoforms per gene) alongside key simulation metrics such as sequencing depth and unique SSC support, providing the foundation for downstream benchmarking analyses.

			Simulated GTF (Transcript Annotation)			Simulated Sequencing Data (Reads)		
**Species**	**Biosample summary**	**Accession**	**Total Genes**	**Total Isoforms**	**Isoforms per Gene**	**Simulated Depth (Read Count)**	**Total Unique SSC**	**Average Read Support per SSC**
Mouse	Left cerebral cortex	ENCFF565RLW	19415	43699	2.3	3M	75382	37
						6M	95188	59
						12M	124202	91
						20M	155173	121
		ENCFF325BXV	18481	39850	2.2	3M	70667	40
						6M	89428	63
						12M	118060	96
						20M	148092	127
	Heart	ENCFF584WWA	15228	30298	2.0	3M	54129	53
						6M	68102	85
						12M	88982	130
						20M	110860	174
		ENCFF860CBL	15727	31563	2.0	3M	56534	51
						6M	71207	81
						12M	93605	124
						20M	117134	165

Supplementary Table 2 | Simulated Data (mouse) Summary Based on Real Transcriptomic Profiles for Benchmarking. This table presents simulated datasets generated by IsoSeqSim using expression profiles derived from real transcriptomic data. It includes transcript annotation complexity (e.g., gene and isoform counts, isoforms per gene) alongside key simulation metrics such as sequencing depth and unique SSC support, providing the foundation for downstream benchmarking analyses.

Dataset Name	Download Link	Sample	Complete GTF (Transcript Annotation)			Sequencing Data (Reads)	
			Total Genes	Total Isoforms	Isoforms per Gene	Total Unique SSC	Average Read Support per SSC
KinnexRelease-UHRR2024-RevioSPRQ	https://downloads.pacbcloud.com/public/dataset/Kinnex-full-length-RNA/DATA-RevioSPRQ-UHRR2024/	FLNC-1	54117	356707	6.6	898319	11.6
		FLNC-2				944449	11.1
		FLNC-3				887981	10.7
		FLNC-4				880023	10.7
		FLNC-5				875404	10.8
		FLNC-6				932939	11.1
SIRV	https://downloads.pacbcloud.com/public/dataset/UHR_IsoSeq/	SIRV	7	61	8.7	3926	25.2

Supplementary Table 3 | Transcriptomic Complexity of UHRR and SIRV Datasets. This table summarizes the annotation complexity and sequencing support of real UHRR and SIRV datasets, including gene and isoform counts, isoforms per gene, the total number of unique SSCs identified from sequencing data, and the average read support per unique SSC.
